# Redox Potential and Antioxidant Capacity of Bovine Bone Collagen Peptides towards Stable Free Radicals, and Bovine Meat Lipids and Proteins. Effect of Animal Age, Bone Anatomy and Proteases—A Step Forward towards Collagen-Rich Tissue Valorisation

**DOI:** 10.3390/molecules25225422

**Published:** 2020-11-19

**Authors:** Laurent Aubry, Claude De-Oliveira-Ferreira, Véronique Santé-Lhoutellier, Vincenza Ferraro

**Affiliations:** INRAE, QuaPA, 63122 Saint-Gènes-Champanelle, France; laurent.aubry@inrae.fr (L.A.); claude.ferreria@inrae.fr (C.D.-O.-F.); veronique.sante-lhoutellier@inrae.fr (V.S.-L.)

**Keywords:** collagen peptides, bovine bone, age, anatomy, antioxidant activity, by-products valorisation

## Abstract

Collagen antioxidant peptides are being widely studied. However, no research has paid attention to biological parameters such as the age and anatomy of collagen-rich tissues, which can determine a change in tissue structure and composition, and then in bioactivity. Moreover, only few research works have studied and assessed peptides antioxidant activity on the food matrix. This work aimed to investigate the effect of bovine’s bone age and anatomy, and of six different enzymes, on the antioxidant activity of collagen peptides. Collagen was extracted from young and old bovine femur and tibia; six different enzymes were used for peptides’ release. The redox potential, the quenching of stable free radicals, and the antioxidant capacity on bovine meat lipids and proteins was evaluated, under heating from ambient temperature to 80 °C. Age and anatomy showed a significant effect; the influence of anatomy becomes most important with age. Each enzyme’s effectiveness toward age and anatomy was not the same. The greatest amount of peptides was released from young bones’ collagen hydrolysed with papain. The antioxidant activity was higher at higher temperatures, except for meat proteins. Assessing the effect of age and anatomy of collagen-rich tissues can promote a better application of collagen bioactive peptides.

## 1. Introduction

Collagen peptides from animal tissues such as skin, tendons, cartilages, bone, etc., have been screened worldwide for numerous biological activities [1,2,3]. Great potential has been shown and in several fields such as food, nutrition, health, cosmetics, materials and biomaterials, sensors, textiles. A broad range of activities has been observed, such as antioxidant, antihypertensive, antidiabetic, anti-inflammatory, immunomodulatory, opioid agonist, wound healing, drug carrier, mineral binding, and aid in synthesising native collagen. Many animal and marine tissues have been considered [1,2,3,4]. While several enzymes and hydrolysis parameters have been tested and optimised, none of the research reported in literature has assessed the effect of some important biological parameters, such as the anatomy of collagen-rich tissues and the age of the animal (or marine) species, on the bioactivity of the collagen peptides. In addition, very few studies have been carried out to assess the potential of bioactive peptides on the food matrix, and those mainly focus on peptides from pork meat and bovine haemoglobin [5]. 

Hence, the objective of this study is to investigate the influence of age and anatomy of a bovine’s bones (from a cow) on the bioactivity of collagen peptides released through six different enzymes, and from three different classes of proteases. Bone has been selected among several collagen-rich tissues because it is the most abundant by-products (in terms of volume and weight) of bovines slaughtering for meat consumption and after milk production [6]; as such, bone can be considered as the most abundant source of collagen, and in particular of type I collagen [2]. The European regulation in force on this category of by-products permits their valorisation in several fields, including food and medicine (article 33 of the EC 1069/2009). Nonetheless, for food applications, bones continue to be converted only into gelatine, however at a low degree (ca. 20%) and with moderate commercial value, or are partially processed into pet-food; even larger quantities are incinerated or exported to limit the disposal costs [7]. This is mainly because no high-value valorisation itineraries have been yet considered and implemented at large extent on the industrial level in Europe despite the sound scientific evidence of the potential of animal by-products such as bone.

This research aims to go beyond the state of the art by supplying novel knowledge regarding the effect of bovine’s bone age and anatomy on the antioxidant activity of collagen peptides, and in particular on their antioxidant power on a food matrix represented by bovine meat. The antioxidant capacity was chosen among many other bioactivities because the oxidative stress is related to the appearance of several diseases such as hypertension, diabetes, obesity, dyslipidaemia, cardiovascular issues, etc. [8,9]. The activity was evaluated as redox potential, quenching of stable free radicals and antioxidant capacity on bovine meat lipids and proteins. The effect of heating from ambient temperature to 80 °C was also considered to assess both the thermal stability of collagen peptides and the effect of temperature on the kinetics of redox potential and radical quenching. 

## 2. Results

### 2.1. Effect of Bone Age and Anatomy, and Enzymes, on the Amount of Collagen Peptides Released

**Figure 1:**
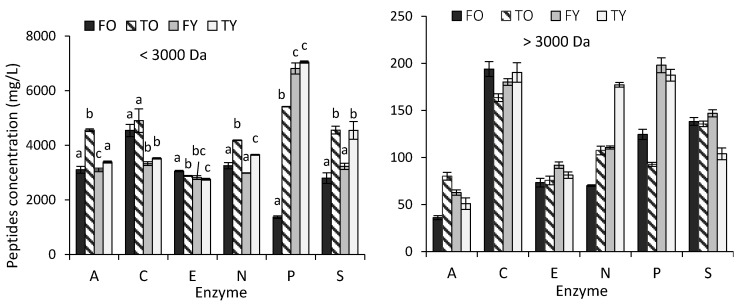
Concentration of peptides, <3000 Da (**left**) and >3000 Da (**right**) (equivalents of collagen, mg/L) from young and old bovine femur and tibia collagen solutions hydrolysed through the enzymes alcalase (A), collagenase (C), esperase (E), neutrase (N), papain (P), savinase (S) (mean values ± standard deviation (SD), *n* = 3). For each enzyme, significant differences among bones (*p* < 0.05) are indicated by different lowercase letters (only for the left Figure). FO = old femur, 10 years; TO = old tibia, 10 years; FY = young femur, 4.5 years; TY = young tibia, 4.5 years.

The amount of collagen peptides varied according to the enzyme used for hydrolysis, and with the bone’s age and anatomy. For the 3000 Da cut-off, the greatest hydrolytic effect was noticed for papain on young bones (Figure 1). If considering as the degree of hydrolysis to 3000 Da the amount of <3000 Da peptides on the total amount of peptides (<3000 and >3000 Da), this was higher than 95% through all the enzymes, whatever the bone’s age and anatomy, unless for FO (old femur, 10 years) hydrolysed with papain, at 92%. The greatest values were obtained with non-specific enzymes (alcalase ≥98%, esperase ≥97%, neutrase and savinase ≥96.5%, for all the bones); with specific enzymes, i.e., collagenase and papain, statistically lower values (95.5% and 96%, respectively) (*p* < 0.05) were found, although the difference was slight.

**Figure 2:**
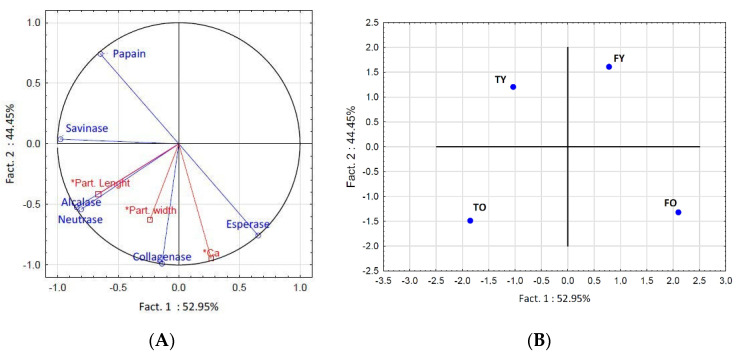
Principal component analysis (PCA) on collagen peptides concentration considering enzymes and particle size as variables (**A**: loading plot) and bone age and anatomy as individuals (**B**: score plot). FO = old femur, 10 years; TO = old tibia, 10 years; FY = young femur, 4.5 years; TY = young tibia, 4.5 years.

PCA (principal component analysis) allowed us to more clearly illustrate and explain Figure 1 data. The two independent principal components (Factor 1 and Factor 2) account for the variability of data at 97.4% and are represented by bone’s anatomy (Factor 1) and bone’s age (Factor 2) (Figure 2). For the bones studied, the anatomy had greater influence than age (52.95% versus 44.45% of data variability explained, respectively) on the amount of peptides released, unless in the case of hydrolysis by collagenase. Moreover, the PCA highlights that with age, the effect of anatomy becomes more significant (Figure 2, score plot). All the enzymes, except esperase, were more active on tibias, where alcalase, collagenase and neutrase were significantly more effective on the old tibia (*p* < 0.05). Moreover, the amount of peptides obtained through collagenase and esperase positively correlated with the amount of calcium, which was higher in old bone peptide solutions.

For all the bones, collagen particle size (diameter) in solution before hydrolysis was in the range ca. 1–35 µm (Figure S1A, in Supplementary Materials), and of same order of collagen in powder, ca. 0.5–15 µm (Figure S1B1, in 1Supplementary Materials), meaning that particle aggregation occurred at a small extent (between ca. 2 and 70 particles), so as the accessibility of the enzyme to the collagen molecules was not (or slightly) affected. Although PCA shows that the variables represented by particle size in solution (Part. Length and Part. Width, Figure 2A) are not as important as the enzyme (loadings are lower) with respect to the amount of peptides released, still there was a strong positive correlation (*r* = 0.91) of particles length with the alcalase and neutrase activity.

The secondary structure of collagen in solution at the optimal hydrolysis temperature for each of the enzymes considered (Table S1 in supplementary data) had no effect on the hydrolytic power of enzymes. PCA showed no correlation with any of the collagen secondary structures and the amount of peptides released by each enzyme, whatever the bone’s age and anatomy; nonetheless, for alcalase and collagenase, a correlation with minor structures was found (Figure S2 in supplementary data).

### 2.2. Antioxidant Activity

**Figure 3:**
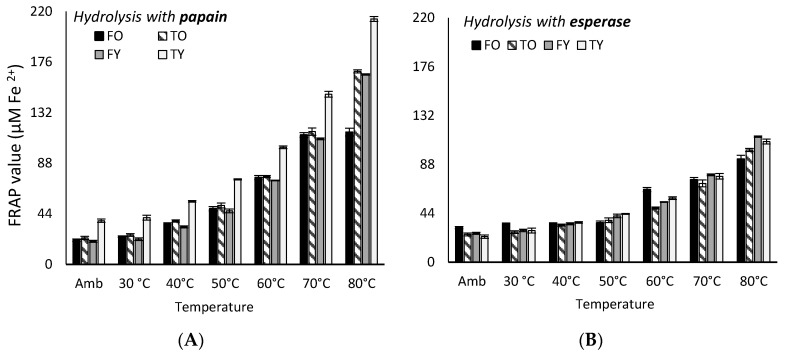
Highest (**A**) and lowest (**B**) FRAP values (µM Fe^2+^) for collagen peptides solutions (<3000 Da) obtained through papain and esperase, respectively, at increasing temperatures. FO = old femur, 10 years; TO = old tibia, 10 years; FY = young femur, 4.5 years; TY = young tibia, 4.5 years.

Collagen peptides showed different antioxidant activity depending on enzymes and the bone’s age and anatomy; the activity not always correlated with peptides concentration. The redox potential, represented by the FRAP (ferric to ferrous iron reducing antioxidant power) value, increased with temperature (*p* < 0.05) (Figure 3); peptides solutions from TY (young tibia, 4.5 years) were the most effective, unless when using esperase. In Table 1 is reported the FRAP index, expressed as mmol of electron donated per 100 g of peptides solution, which varied according to enzyme used and in the order papain > alcalase > collagenase > neutrase > savinase > esperase (*p* < 0.05). However, when considering the relative amount of peptides in each solution, enzyme contribution on the FRAP index was different and in the order alcalase > collagenase > esperase > neutrase > papain > savinase (Table 2); alcalase TY peptides compared with papain FO peptides. No statistical differences (*p* > 0.05) were noticed among FRAP values after 30 and 180 min of incubation.

**Figure 4:**
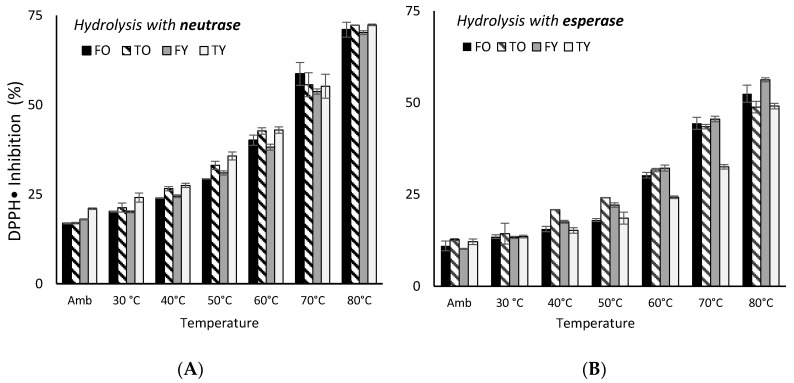
Highest and lowest DPPH• inhibition (%) for collagen peptides solutions (<3000 Da) obtained through neutrase (**A**) and esperase (**B**), respectively, and at increasing temperatures. FO = old femur, 10 years; TO = old tibia, 10 years; FY = young femur, 4.5 years; TY = young tibia, 4.5 years.

**Figure 5:**
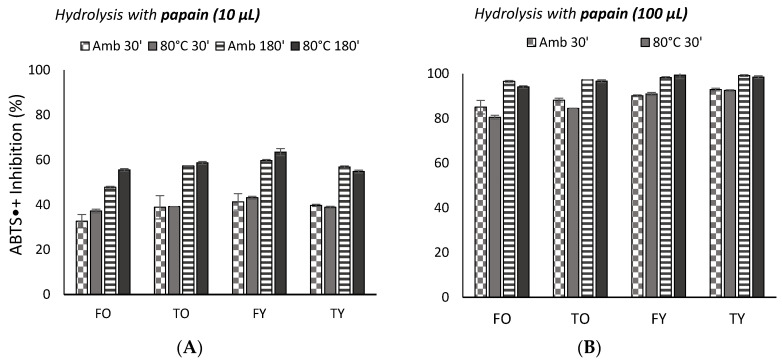
ABTS•+ inhibition (%) by collagen peptides solutions (<3000 Da) obtained through the most effective enzyme, i.e., papain, for 10 µL (**A**) and 100 µL peptides solutions (**B**), at ambient temperature and 80 °C, and after 30 ant 180 min of incubation. FO = old femur, 10 years; TO = old tibia, 10 years; FY = young femur, 4.5 years; TY = young tibia, 4.5 years.

Inhibition of DPPH• (2,2′-dyphenil-1-picryl-hyrazyl radical) by peptides solutions also increased with temperature and enzyme contribution was in the order neutrase > alcalase > papain > savinase > collagenase > esperase (*p* < 0.05) (Figure 4) when comparing the same volume of peptides solutions. For the most effective enzyme, >70% DPPH• inhibition was noticed at 80 °C after 30 min of incubation, whatever the age and the anatomy. At T <60 °C, on the contrary, tibia peptides showed highest activity (*p* < 0.05) (Figure 5). Although the less effective, peptides from savinase still induced ≥50% radicals inhibition at 80 °C, where femurs peptides showed the highest activity (*p* < 0.05). No statistical differences (*p* > 0.05) were found among inhibition after 30 and 180 min of incubation.

When comparing the DPPH• inhibition per mg of peptides, FO peptides were the most effective and enzyme contribution was in the order papain > neutrase > alcalase > savinase > esperase > collagenase (Table 3).

**Figure 6:**
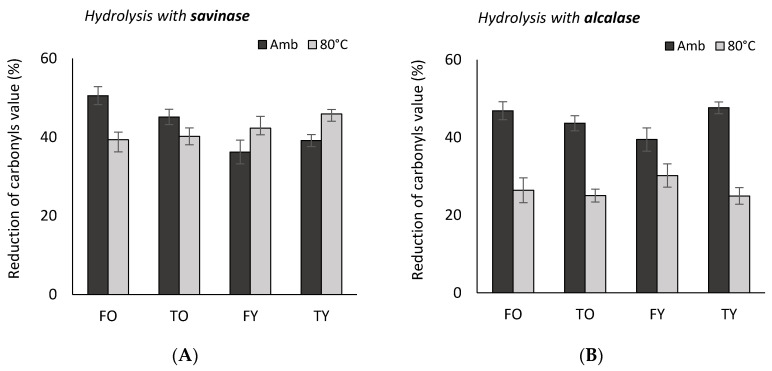
Reduction of carbonyls value (%) in bovine meat (semimembranosus muscle) at ambient temperature and at 80 °C through the addition of collagen peptides solutions (<3000 Da) from young and old bovine femur and tibia obtained through the enzymes savinase (**A**) and alcalase (**B**). FO = old femur, 10 years; TO = old tibia, 10 years; FY = young femur, 4.5 years; TY = young tibia, 4.5 years.

Inhibition of ABTS•^+^ (2,2′-azinobis-*3*-ethyl-benzothiazoline-*6*-sulfonic-acid radical cation) increased significantly (*p* < 0.05) with the incubation time and with temperature (Figure 5). By the addition of the 100 µL sample, the inhibition was higher than 80% and up to 100% at ambient temperature and 80 °C. To overcome a probable effect of saturation, an assay with 10 µL was also performed and results showed that, after 180 min of incubation at 80 °C, the ABTS•^+^ inhibition was the highest (*p* < 0.05) and higher than 55%. When comparing the same volume of peptides solutions, FY (young femur, 4.5 years) peptides were the most effective (*p* < 0.05) (63% inhibition, Figure 6A); for all the bones, enzymes’ contribution was in the order papain > collagenase > alcalase > savinase > esperase > neutrase (*p* < 0.05. When comparing the same amount of peptides, those from FO hydrolysed with papain were the most effective, followed by FY peptides obtained by collagenase; neutrase was once again the less effective protease.

Collagen peptides also showed the capacity to reduce carbonyls’ value in bovine meat. Without the addition of collagen peptides the carbonyls value was 6.3 ± 0.8 nM hydrazones/mg protein (*n* = 6), which corresponds to a significant oxidative status (>3 nM hydrazones/mg protein) [10]. When adding peptide solutions, the highest antioxidant activity (51–43% of carbonyls value reduction) was noticed by FO peptides and in the enzymes order savinase > papain > neutrase ≥ collagenase ≥ alcalase > esperase (*p* < 0.05); the lowest activity was observed by FY peptides (43–35%). The antioxidant power was always higher at ambient temperature with respect to 80 °C, except for FY and TY (tibia young, 4.5 years) peptides obtained by savinase (*p* < 0.05) (Figure 6A); moreover, by this enzyme, the loss of antioxidant activity with temperature was the smallest, whatever the age and the anatomy of bones. By contrast, peptide solutions obtained by alcalase showed the highest loss of antioxidant power when increasing temperature (Figure 6B). In Table 4 are reported the carbonyls values reductions (%) per mg of peptides, and relative to 210 mg of meat protein, i.e., 1 g of the raw meat considered (according to the assay described in Section 4.5.2).

**Figure 7:**
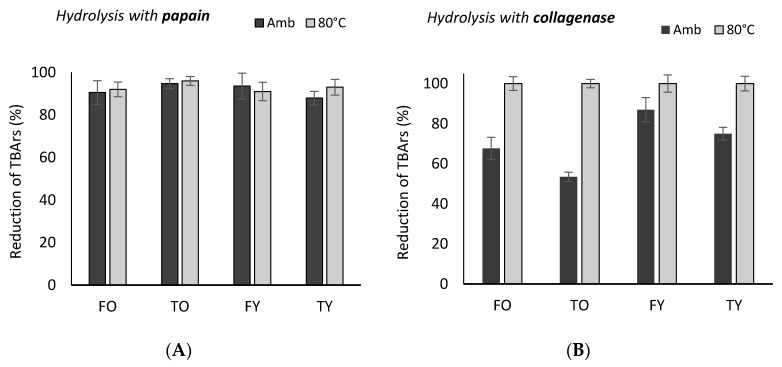
Reduction of TBArs (%) in bovine meat (semimembranosus muscle) at ambient temperature and at 80°C through the addiction of collagen peptides solutions (<3000 Da) from young and old bovine femur and tibia obtained through the enzymes papain (**A**) and collagenase (**B**). FO = old femur, 10 years; TO = old tibia, 10 years; FY = young femur, 4.5 years; TY = young tibia, 4.5 years.

Collagen peptides were able to reduce lipid oxidation, i.e., TBArs, (thiobarbituric acid reactive substances) and their activity was higher at 80 °C. Without the addiction of collagen peptides the amount of TBArs was 1.1 ± 0.13 mg MDA (malondialdehyde)/kg meat (*n* = 6), which corresponds to a significant oxidative status of lipids (>0.5 mg MDA/kg meat) [11]. Enzymes effect was different and in the order papain > savinase > neutrase > esperase > alcalase > collagenase (*p* < 0.05). For the most effective enzyme, although TO peptides showed the highest activity (96% of carbonyls reduction), there probably was a saturation effect due to a high peptides concentration in the assay; a reduction higher than 90% was in fact noticed, and with no statistical difference between ambient temperature and 80 °C (Figure 7). Besides, when using papain, the effect of age and anatomy was not evident. When using collagenase, by contrast, age, anatomy and temperature carried significant different responses: peptides from tibia were more effective than those from femur as well as young bones peptides (*p* < 0.05); by contrast with the carbonyl values, TBArs reduction increased at 80 °C and up to 100% through all the enzymes (Figure 7). In Table 5 are reported the TBArs’ value reductions (%) per mg of peptides, and relative to 22.5 mg of meat lipids, i.e., 1 g of the raw meat considered (according to the assay described in Section 4.5.2).

**Figure 8:**
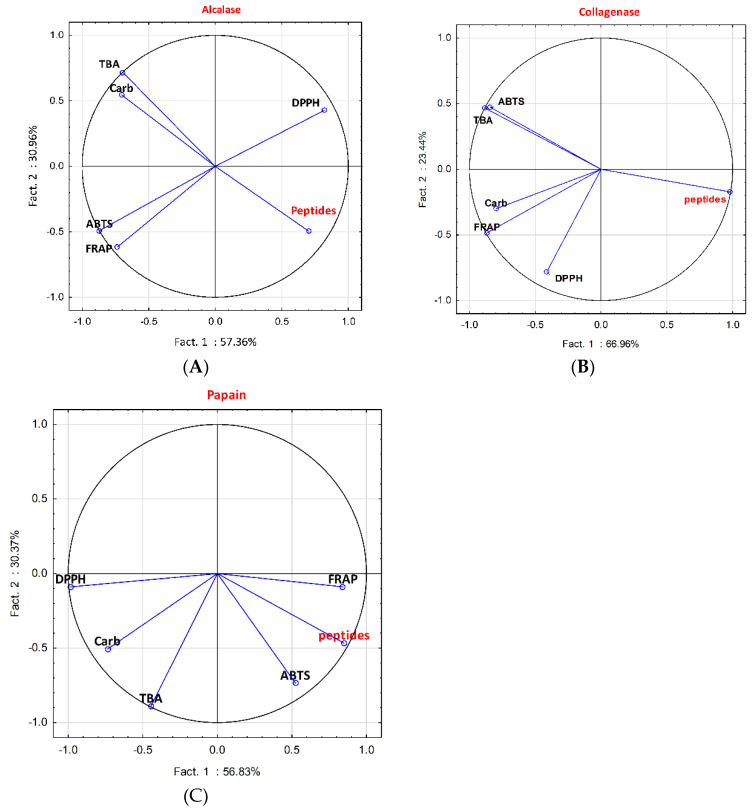
PCA loading plot of antioxidants activity and peptides concentration for alcalase (**A**), collagenase (**B**) and papain (**C**).

According to the enzyme used, antioxidant activities did not correlate in the same way with peptides’ concentration; antioxidant activities also correlated differently depending on the enzyme. Considering, for instance, three proteases from different classes (see Section 4.2), i.e., alcalase, collagenase and papain, amount of peptides strongly negatively correlated (*r* < −0.85) with TBArs reduction in the case of alcalase, it correlated negatively with TBArs reduction and ABTS•^+^ inhibition for collagenase, and positively (*r* > 0.85) with FRAP values for papain (Figure 8). When using alcalase, ABTS•^+^ inhibition correlated positively with FRAP value and negatively with DPPH• inhibition; TBArs and carbonyls positively correlated with each other (*r* > 0.85). In the case of collagenase, correlations (all positives, *r* > 0.85) were found between ABTS•^+^ inhibition and TBArs reduction, and between FRAP values and carbonyls reduction. For papain, there was no significant correlation of peptides concentration for any of the antioxidant activities with each other.

**Figure 9:**
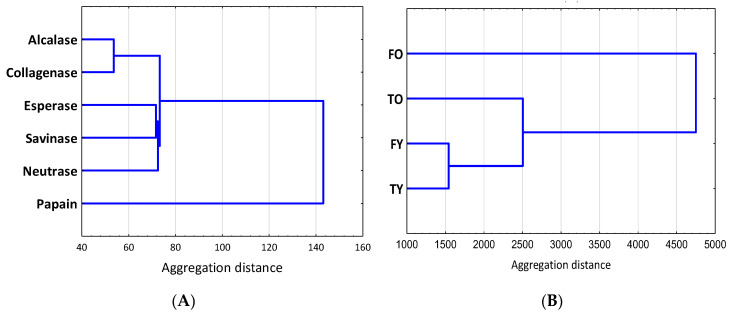
(**A**) Hierarchical clustering analysis (HCA) of enzymes on the basis of the antioxidant activities and for all the bones (FO, TO, FY and TY); (**B)** HCA of bones based on antioxidants activities and amount of peptides released by each enzyme. FO = old femur, 10 years; TO = old tibia, 10 years; FY = young femur, 4.5 years; TY = young tibia, 4.5 years.

**Figure 10:**
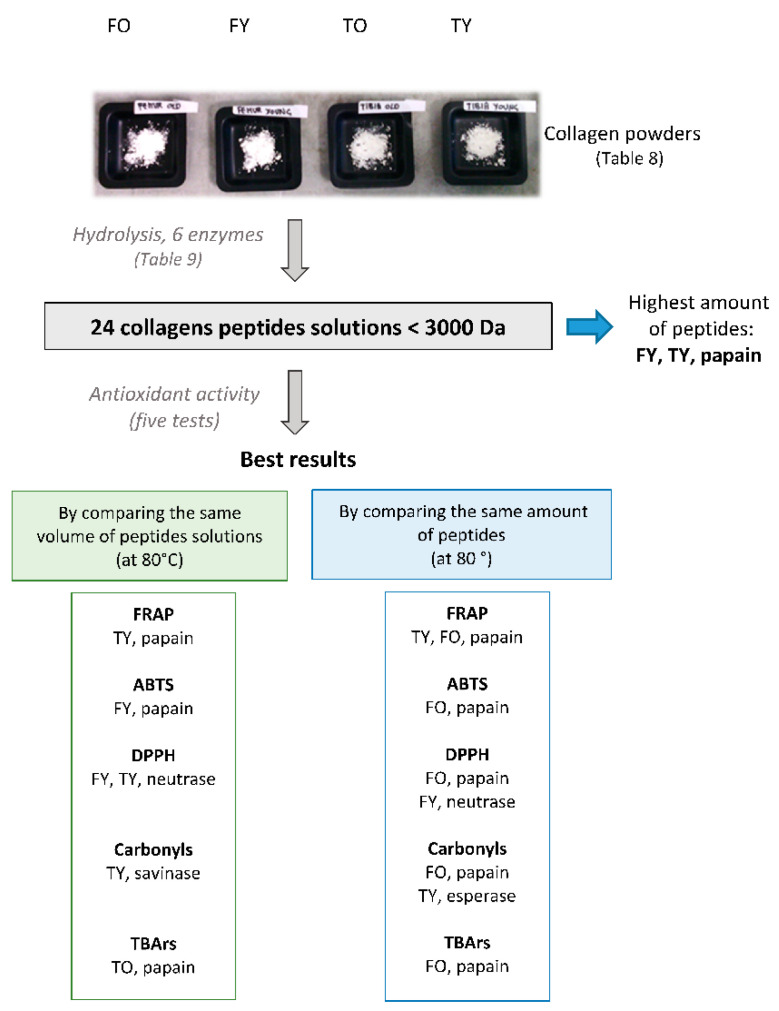
Overview of the antioxidant activity of bovine bone collagen peptides (at 80 °C) by considering bones of different ages and anatomies (femur old, 10 and; femur young, 4.5 years; tibia old, 10 years; tibia young, 4.5 years), and six enzymes (alcalase, collagenase, esperase, neutrase, papain, savinase).

By considering all the bones studied, a HCA (hierarchical clustering analysis) (Figure 9) showed that alcalase and collagenase peptides had the most similar antioxidant activities (considering all the tests performed), as well as the group represented by esperase, savinase and neutrase; moreover, esperase and savinase, both from *Bacillus lentus* and with the same molecular weight, belong to the same cluster. By contrast, peptides obtained through papain acted in a different way. HCA performed bone by bone led to the same conclusion (results not shown). When considering the antioxidant activities and the amount of peptides from each bones, FO and TY showed the largest difference while FY and TY the most similar effect (they belong to the same cluster) (Figure 10A). The results of HCA also confirmed the PCA finding that with age the effect of the anatomy become more important (the distance between FO and TO is higher than the distance between FY and TY) (Figure 10).

### 2.3. Kinetics of FRAP and DPPH Assays Antioxidant Activity Under Heating

Kinetics of the redox potential, based on the iron reducing activity (FRAP assay) by collagen peptides, is well represented by one-phase exponential growth function all over the temperature range considered (Equation (1a) and (1b)), unless for FO peptides either in the case of papain (more effective enzyme) or esperase (less active enzyme), for which two kinetics occurred. For the papain FO peptides, the one-phase exponential model (Equation (1a)) fitted form ambient temperature to 70 °C, after which it was linear and slower up to 80 °C (Equation (2)) (two intermediate T values—73 and 77 °C—were studied to perform the fitting). In the case of esperase FO peptides, the kinetics followed a logarithmic model (Equation (3)) from ambient temperature to 50 °C, and the faster exponential model (Equation (1b)) from 50 to 80 °C.

(1a) $$y\;=\;y_{0}+A\cdot \;e^{T/t}$$

(1b) $$y\;=\;y_{0}+A\cdot \;e^{\left(T-T0\right)/t}$$

(2) y=a+b·T

(3) y=ln(a+b·T)

where *y* is the FRAP value at a specific temperature *T*, *T_0_* is the initial temperature, *y*_0_ is the FRAP value at the infinitely small temperature (T = −∞) and the other terms are constants. The kinetic parameters relative to the hydrolysis with papain and esperase are reported in Table 6.

In the case of DPPH• quenching activity, when considering the more and less effective enzyme (Figure 5), the one-phase exponential growth function (Equation (1a)) fitted data for TO, FY and TY peptides obtained by neutrase, and TY peptides obtained by esperase. For the other peptides solutions, the temperature of 50 °C was a critical value: it determined a change in the kinetics, which became faster afterward. For the esperase FO peptides, two logarithmic models were found:

(4) y=a−b·ln(T+c) ambient ≤ T ≤ 50 °C

(5) y=a ·ln(−b·ln(T)) T > 50 °C

Two linear kinetic models (Equation 2) occurred for TO and FY peptides obtained by esperase, while in the case of FO neutrase peptides, the model was linear up to 50 °C (Equation (2)) and logarithmic after (Equation (5)). For the DPPH kinetics, *y* is the percentage of DPPH• inhibition at the temperature *T*, *y*_0_ is the DPPH• percentage inhibition at the infinitely small temperature (T = −∞). The kinetics parameters are reported in Table 7. 

## 3. Discussion

### 3.1. Effect of Bone Age and Anatomy, and Enzymes, on the Amount of Collagen Peptides Released

The amount of peptides released from collagen extracted from bovine bones, is influenced by bone’s age and anatomy, for each enzyme considered. The results mainly reflect the changing structure of the bone’s organic and mineral phase with age and anatomy, the changing composition with respect to some minor compounds (such as the non-collagen molecules) and the changing ratio of the characteristic bone elements Ca and P [12,13,14]. All these factors affected the composition of collagen solutions (Table 8). Nevertheless, regarding age, attention should be paid to the evidence that this study focuses on quite extreme values, i.e., 4.5 and 10 years, which correspond to young and old bovines, respectively. For each enzyme used, the extent of the influence of age and anatomy on the amount of collagen peptides was not the same (Figure 1). Regarding subtilisins, esperase and savinase, although derived both from *Bacillus lentus* and with the same molecular weight (see Section 4.3), they showed a different selectivity towards age and anatomy (Figure 1), which highlights the influence of the bacterial strain from which they are produced. Collagenase is a characteristic protease of bones, where it promotes the remodeling with age (and also with weight and/or pathological conditions) [12,13]; it was shown to be more effective on femurs collagen, and with slight differences between ages, which can be probably due to the lower amount of non-collagenous molecules and the higher amount of minerals with respect to tibias (Table 8). Papain showed the highest hydrolytic effect, which is probably in a link with his specificity with respect to subtilisins, and to different cutting sizes with respect to collagenase (Table 9). The mineral phase probably influenced the papain hydrolytic power; the enzyme was in fact more effective on young bones and less effective on old femur. However, when considering the action of the enzymes on the collagen hydrolysis, it should be taken into consideration that di-peptides (and free amino acids) are not included in this analysis, since the method used for peptides quantification demands at least a tri-peptide, as all other colorimetric methods [15]. Further studies will be carried out on the quantification, by chromatographic methods, of di-peptides and free amino acids released from collagen hydrolysed with the enzymes considered. 

### 3.2. Antioxidant Activity 

Bovine bone collagen peptides, whatever the age and anatomy, showed a significant redox potential (represented by the iron reducing capacity) as well as the ability to quench stable free radicals, ABTS•^+^ and DPPH•, at a large extent, and to reduce carbonyl and TBArs values in bovine meat to an important degree. For the redox potential, all peptides compare with the 15 foods having the highest FRAP index (FI), which are of plant origin, and, in particular, spices and herbs [16]. TY alcalase peptides and FO papain peptides, the more active (Table 2), compare with dried oregano leafs (FI 40.3), only second to ground cloves (FI 125.5) which have the highest redox potential among 1130 foods tested; FO collagen peptides, the less active, compare with dried parsley (FI 7.4) [17]. Among 3139 foods of different categories studied, Carlsen et al. [16], showed that plant-based foods are generally higher in antioxidant power (FI) than animal-based and mixed foods. The results of our study can, therefore, corroborate the finding that animal proteins, such as collagen, can release peptides with higher antioxidant activity then the protein itself [4,18] and those can well compare with plant origin food with respect to the antioxidant power. 

Regarding the quenching of the free radicals ABTS•^+^ and DPPH•, although these are only partially representative of biological molecules, the results can give useful information on the ability of all the collagen peptides studied to transfer a hydrogen atom or an electron towards whichever radical in order to neutralise it [19,20]. Also, as ABTS•^+^ and DPPH• are much more inert than many other synthetic and biological radicals (they persist in solution for long minutes to hours, by contrast with biological radicals which have lifetimes of seconds) [19], the results of this study highlight the capability of collagen peptides to quench very stable radicals. Results from the DPPH assay allow evaluating the power of collagen peptides to neutralise reactive oxygen species (ROS) such as peroxyl radicals (ROO•), alkoxyl radicals (RO•), superoxide anion (O_2_^−^), hydrogen peroxide (H_2_O_2_) and hydroxyl radical (HO•), quenched with the same mechanism of DPPH• (hydrogen atom transfer mainly); among all ROS, ROO• has the higher biological relevance and the longest half-life [19]. Moreover, the DPPH assay is representative of the antioxidant capacity towards lipophilic radicals [19,20]. The ABTS test, although not suitable for simulating ROS neutralisation (since ABTS•^+^ is a larger and sterically hindered radical centred on nitrogen instead of oxygen), can be representative of aromatic nitrogen radicals quenching [19]. Its simple implementation allows us to easily compare interrelated classes of samples, as in the case of our study, and can reflect changes in biological and food samples that undergo treatments/processing (heating, drying, etc.) [21]. Moreover, as the ABTS•^+^ radical is soluble in both organic and aqueous solvents [19], our results show that collagen peptides can quench both hydrophilic and lipophilic radicals. Another interesting aspect to highlight is that the ABTS•^+^ chemical structure, represented by two symmetric aromatic heterocyclic groups (ethylbenzothiazoline) one of which carries the nitrogen radical, is very similar to the neutral tryptophan indolyl radical (TprN•). This radical participates in different radical pathways and in particular in the oxidation of tyrosine, an amino acid with major functions in the human body [22]; as such, our results could likely give information about the ability of collagen peptides to quench such a radical. Further studies will be carried out to investigate this subject. 

When tested on a biological sample, represented by bovine meat, all the collagen peptides showed a significant activity in preventing either proteins or lipids oxidation. Oxidative modifications of proteins can occur in numerous processes and by different agents (oxygen radicals, redox cations such as Fe^2+^ and Cu^2+^, products of lipid oxidation, etc.). The appearance of carbonyl groups (aldehydes and ketones) is considered an early marker of oxidative modification of proteins, where lysine, arginine, proline and histidine are the most likely amino acids to form carbonyls derivatives [23]. Although the antioxidant activity of collagen peptides was lower at 80 °C with respect to the ambient value, the results show that at least 25% or carbonyl value reduction is possible (by the less active peptides, Figure 7B) when cooking meat muscle in the presence of collagen peptides, at 80 °C and up to 30 min. By contrast, the antioxidant activity towards lipids peroxidation was higher at higher temperature, which can bring a significant contribution to the nutritional quality of cooked meat. Lipid peroxidation is relative to the oxidative deterioration of lipids containing carbon–carbon double bonds as in the case of unsaturated fatty acids (including ω3 and ω6), phospholipids, glycolipids, etc., and which can be easily evaluated by the TBArs essay [24]. Malondialdehyde (MDA), the main product of unsaturated lipid oxidation, can form adducts with food proteins with a negative effect on their nutritional value [11,19,25]. From a biological point of view, MDA levels increase with age in the human body; in addition, MDA-proteins and MDA-DNA adducts can give rise to pro-inflammatory and pro-fibrogenic effects. Higher levels of MDA have been also found in patients affected by Parkinson’s disease with respect to healthy individuals [25]. These findings suggest that the antioxidant activity of bovine bone collagen peptides could be also tested in aging and in such disease status. 

The results also showed that the redox potential and the antioxidant activities does not always correlate with the amount of peptides. This highlights that di-peptides and free amino acids (disregarded in this study), as well as peptide composition, can make a crucial contribution to the antioxidant power, as also highlighted by previous research [4]. Further investigations will be carried out by the authors on this topic. 

### 3.3. Kinetics of FRAP and DPPH Antioxidant Activity Under Heating 

The analysis of the kinetics of the redox potential under heating allowed highlighting that, when using papain, the most effective enzyme, young bones peptides were the most reactive; on the contrary, old femur peptides were the most reactive ones when using esperase, the less effective enzyme (Figure 4, Table 6). The analysis of the DPPH• quenching kinetics allowed to show that the temperature of 50 °C is a critical value (Figure 5, Table 7); it determines a change in the kinetic model with a faster increase of the antioxidant activity with temperature. This could be likely due to an increase in the kinetic energy of the systems, and then in the speed of species in solution, which favoured the antioxidant reactions. 

### 3.4. Summary and Future Research 

This study compared four bovine leg’s bones—two anatomies (femur and tibia) and two biologically distinct ages (young and old)—with respect to the antioxidant activity of <3000 Da collagen peptides. Collagen was extracted with the same methodology for all the bones, and this led to four different extracts with respect to collagen, non-collagen and mineral concentration (Table 8). The amount of protease used for the hydrolysis was normalised on the concentration of collagen; 24 solutions were then obtained by hydrolysing the four bones collagen solutions with the six different enzymes selected. All the <3000 Da peptide fractions showed a significant antioxidant activity through all the five assays considered (FRAP, ABTS, DPPH, carbonyls and TBArs). Although the highest amount of <3000 Da peptides was obtained by the hydrolysis of FY and TY with papain, the antioxidant activity did not correlate with the peptides amount. Still, papain globally appeared as the best protease. An overview of the results in reported in Figure 10. Further studies will be carried out to assess the effect of the amount of di-peptides and free amino acids, as well as of the compositions of the peptides on the antioxidant activity observed. Moreover, the effect of the mineral phase on the antioxidant activity will be screened more in depth by studying the mineral-free solutions. 

## 4. Materials and Methods

### 4.1. Bone Samples and Extraction of Collagen 

Collagen was extracted from 4 different bovine bones, accounting for 2 different ages and 2 different anatomies, namely femur and tibia from a young Prim’Holstein cow (4.5 years) (hereafter FY and TY, respectively) and femur and tibia from an old Prim’Holstein cow (10 years) (hereafter FO and TO, respectively). Bones were collected at the experimental slaughterhouse of INRAE (*Institut Nationale de Recherche pour l’Agriculture, l’Alimentation et l’Environnement*—National Research Institute for Agriculture, Food and the Environment) (Saint-Genès-Champanelle, France). Extraction of collagen and drying of collagen solutions was carried out as described in Ferraro et al. [12]. Composition of powders is reported in Table 8.

### 4.2. Scanning Electron Microscopy of Collagen Powders, and Fourier Transform Infrared (FTIR) Spectroscopy and Particle Size Analysis of Collagen in Solution 

Collagen powders from FO, TO, FY and TY were observed with the equipment Quanta FEG (Field Emission Gun) 250 (FEI, Mérignac, France) at the accelerating voltage of 15 kV and 5000× magnification. Before hydrolysis, collagen powders were dissolved in 0.1 mM acetic acid at 3 mg/mL and the infrared spectra were acquired at different temperatures, corresponding to the optimal value for each enzyme (Table 9). The FTIR Tensor II (Bruker; Villeurbanne, France) spectrometer equipped with a cell specially created to analyse proteins in solution (*AquaSpec*^®^, Bruker) was used. Secondary structure of collagen from each bone and at different temperatures was obtained through the analysis of the second derivative in the Amide I (1600–1700 cm^−1^) spectral zone. Particle size dimension for the same collagen solutions was analysed with the equipment Sysmex FPIA-3000 (Malvern, Villepinte, France) (1–400 µm particle diameter range). 

### 4.3. Hydrolysis of Collagen

Collagen from FO, TO, FY and TY was hydrolysed by 6 different proteases, all endopeptidases (Sigma-Aldrich, Saint-Quentin Fallavier, France): collagenase B, 68–130 kDa (a metalloprotease, from *Clostridium histolyticum*), papain, 23.4 kDa (a cysteine protease, from the latex of papaya (*Carica papaya*)), and 4 different subtilisins (serine proteases), namely alcalase, 20–45 kDa (from *Bacillus licheniformis*), neutrase, 37 kDa (from *Bacillus amyloliquefaciens*), esperase and savinase, 20–30 kDa (subtilisin 147 and 309, respectively, both from *Bacillus lentus*) [26]. The hydrolysis was carried out for 24 h under gentle stirring, at the optimal pH and temperature conditions for each enzyme (Table 9) and at the ratio of collagen to enzyme 1:20 by weight. Collagen powders from FO, TO, FY, and TY were dissolved in 0.1 mM absolute acetic acid (Sigma-Aldrich, Saint-Quentin-Fallavier, France) first and the pH was then adjusted to the optimal value with 1 M NaOH (Sigma-Aldrich). After hydrolysis, enzymes were inactivated by heating at 95 °C for 5 min; a cut-off of 3000 Da (Merk Millipore, Guyancourt, France) was applied though centrifugation at 5000 rpm for 45 min at ambient temperature. The permeate solutions were considered for the screening on the antioxidant activity.

### 4.4. Collagen Peptides Quantification

Collagen peptides concentration was determined with the bicinchoninic acid (BCA) test, as described by Smith et al. [15], and using the BCA assay kit developed by Sigma-Aldrich. The BCA reagent was daily prepared by adding 50 parts of reactive A (aqueous solution of 1% BCA-Na_2_, 2% Na_2_CO_3_ · H_2_O, 0.16% Na_2_ tartrate, 0.4% NaOH and 0.95% NaHCO_3_ by weight) to 1 part of reactive B (4% CuSO_4_ · 5H_2_O by weight in deionised water). A volume of sample of 100 µL was added to 2 mL of the BCA reagent and the solution was incubated at 60 °C for 30 min then cooled to the ambient temperature for 5 min using a water bath. The absorbance was read straight away, at 562 nm, using a Jasko V-770 spectrophotometer (Jasko; Lisses, France). Absorbance values for samples were standardised against the absorbance of rat-tail collagen type I (Sigma-Aldrich) solutions in the range 5–2500 mg/L. All the determinations were done in triplicate. 

### 4.5. Antioxidant Activity and Effect of Heating 

Antioxidant activity of < 3000 Da collagen peptides solutions was evaluated by 5 different tests, 3 of which based on cell-free synthetic radical systems (ABTS, DPPH and FRAP) and 2 of which (TBArs and carbonyls) carried out on bovine meat (semimembranosus muscle from Charolaise heifer obtained from a supermarket). Assays are described in detail below.

#### 4.5.1. ABTS, DPPH and FRAP Assays

The ABTS test was performed with the method reported by Guimarães et al. [27]. The peroxyl radical ABTS•^+^ was generated by mixing 7 mM ABTS (2,2′-azinobis-*3*-ethyl-benzothiazoline-*6*-sulfonic-acid) (Sigma-Aldrich) in ultrapure water with 2.54 mM potassium persulfate (Sigma-Aldrich) in ultrapure water, in the proportion 1:1 (*v*/*v*); the mixture was then allowed to react during 16 h in the dark and at a room temperature. The working solution of ABTS•^+^ was prepared daily by diluting the stock solution in ultrapure water so as the absorbance at 734 nm was in the range 0.700 ± 0.20, measured on a Jasko V-770 spectrophotometer. For each peptide solution, two sample volumes were tested, 10 and 100 μL, added to 1 mL of the working ABTS•^+^ solution. The absorbance at 734 nm was read after 30 min and up to 180 min each half-hour, at ambient temperature and at 80 °C, with incubation in the dark. A blank sample of ultrapure water was also analysed at the same conditions. Results are expressed as percentage of inhibition, *I* (%), of ABTS•^+^ as follows:

(6) $$I\left(\%\right)=\;\frac{\left(Abs_{ABTS•+}-\;Abs_{Sample})-\;(Abs_{ABTS•+}-\;Abs_{Blank}\right)}{\left(Abs_{ABTS•+}-\;Abs_{Blank}\right)}\;\times \;100$$

where *Abs_ABTS__•+_* is the absorbance of ABTS•^+^, and *Abs_Sample_* and *Abs_Blank_* are the sample and blank absorbance, respectively. All determinations were done in triplicate.

DPPH test was performed according to Brand-Williams et al. [28]. A stock solution of 600 µM 2,2′-dyphenil-1-picryl-hyrazyl radical (DPPH•) (Sigma-Aldrich) was prepared by adding 24 mg to 100 mL absolute ethanol (Sigma-Aldrich), and stored at −20 °C until needed. The working solution was prepared daily by diluting the stock solution with absolute ethanol to 60 µM DPPH•, and had an absorbance of 0.670 ± 0.20 at 517 nm, measured on a Jasko V-770 spectrophotometer. A volume of sample of 300 μL was added to 2.7 mL of DPPH• working solution and absorbance at 517 nm was read after 30 and 180 min of incubation and at different temperatures, namely ambient (ca. 22 °C), 30, 40, 50, 60, 70 and 80 °C, with incubation in the dark. A blank sample of ultrapure water was also analysed at the same conditions. Results are expressed as percentage inhibition, *I* (%), of DPPH• as follows:

(7) $$I\left(\%\right)=\;\frac{\left(Abs_{DPPH•}-\;Abs_{Sample}\right)-\;\left(Abs_{DPPH•}-\;Abs_{Blank}\right)}{\left(Abs_{DPPH•}-\;Abs_{Blank}\right)}\;\times \;100$$

where *Abs_DPPH__•_* is the absorbance of DPPH•, and *Abs_Sample_* and *Abs_Blank_* are the sample and blank absorbance, respectively. All determinations were done in triplicate.

FRAP (ferric to ferrous iron reducing antioxidant power) assay was performed to evaluate the redox potential of collagen peptides and was carried out with the method described by Benzie and Strain [29]. Acetate buffer 300 mM pH 3.6, TPTZ (2,4,6-tripyridyl-*s*-triazine) 10 mM in 40 mM HCl, and FeCl_3_ · 6H_2_O 20 mM (Sigma-Aldrich) were mixed in the proportion 10:1:1 to prepare the working FRAP reagent daily, where the final concentration of Fe^3+^ is 1.67 mM. Aqueous solutions of known concentration of Fe^2+^ (from FeSO_4_·7H_2_O, Sigma-Aldrich) in the range 100–800 μM were used for calibration; acetate buffer and TPTZ were added in the same proportion as for the FRAP reagent (10:1:1). For each calibration point, a volume of 3 mL was used and the absorbance was read at 593 nm after 30 min of incubation in the dark. Peptides solution volume was 0.3 mL, added to 2.7 mL FRAP reagent and absorbance reading at the same wavelength after 30 min and 180 min of incubation and at different temperatures, namely ambient, 30, 40, 50, 60, 70 and 80 °C. Solutions used for calibration were incubated at the same temperatures as the samples. The redox potential was expressed as FRAP value, i.e., µM Fe^2+^ (obtained from calibration lines), and as FRAP index, i.e., mmol of electrons donated per 100 g of peptides solutions and per 100 g of peptides, for each enzyme used.

#### 4.5.2. TBArs and Carbonyls Determination 

The TBArs test, used to assess lipids peroxidation, was performed according to the method developed by Lynch and Frei [11]. 1 g of raw meat muscle (4.5% lipids on raw weight) was finely ground in 9 mL phosphate buffer (pH 7.4) in an ice bath to avoid oxidation. TBArs values were determined for raw bovine meat muscle without the addition of collagen peptides (blank) and with the addition of collagen peptides, at ambient temperature and at 80 °C. For the blank test, 500 µL of ground meat were incubated with 500 µL of water, 1% (*w*/*v*) 2-thiobarbituric acid in 50 mM NaOH (0.25 mL) and 2.8% (*w*/*v*) trichloroacetic acid (0.25 mL) in a boiling water bath for 10 min. After cooling to room temperature the pink chromogen was extracted into *n*-butanol (2 mL) and its absorbance at 535 nm was measured. All reagents were from Sigma-Aldrich. To assess the effect of collagen peptides, 500 µL of ground meat were incubated with 500 µL of each peptides solution, and the assay was carried out as above. TBArs concentrations were calculated as equivalents of malondialdehyde (MDA) (mg MDA/Kg of meat), the most prominent product of lipids peroxidation, through its molar extinction coefficient which is 1.56 × 10^5^ M^−^^1^·cm^−^^1^. The percentage of reduction of TBArs due to the addition of collagen peptides was then calculated with respect to TBArs values without peptides.

The carbonyls assay, used to evaluate protein oxidation, was carried out with the method described by Oliver et al. [10]. Carbonyl values were determined for meat without the addition of collagen peptides and for meat with the addition of collagen peptides, at ambient temperature and at 80 °C. 1 g of raw meat muscle (21% protein on raw weight) was ground in 1 mL of water and 9 mL KCl 0.15 M containing 0.1 mM Fe^2+^ and 0.1 mM H_2_O_2_ to induce oxidation. To evaluate the effect of collagen peptides, 1 g of meat was grinded with 1 mL of peptides solution and 9 mL KCl 0.15 M containing 0.1 mM Fe^2+^ and 0.1 mM H_2_O_2_ to induce oxidation. Two aliquots of 300 µL of ground meat solution and two aliquots of 300 µL of ground meat solution with peptides were both incubated at ambient temperature and at 80 °C for 30 min. After incubation all the aliquots were then treated with 10% *w*/*v* trichoroacetic acid (TCA) and centrifuged for 10 min at 4 °C and 4000 rpm; the supernatants were discarded afterward. For each group of aliquots, one precipitate was mixed with 1 mL HCl 2 N, and the other with 1 mL DNPH 0.2% (*w*/*v*) in HCl 2 N. Both fractions were stirred for 60 min at ambient temperature in the dark and precipitated again with 10% TCA. A second centrifugation at the same conditions was then carried out and the precipitates were washed three times with 1 mL ethanol/ethyl acetate (1/1 *v*/*v*) to eliminate lipids and excess DNPH. Precipitates were then dissolved in 2 mL guanidine-HCl 6 M in phosphate buffer 20 mM pH 6.5, stirred during 2 h and finally centrifuged for 10 min at 4000 rpm. Absorbance of aliquots treated with DNPH was read at 360 nm and absorbance of aliquots without DNPH was read at 280 nm. The concentration of carbonyls, expressed as nanomoles of hydrazones/mg protein, was determined as (*Abs_370_ · 0.65*)/(*Abs_280_* · *21*) where 21 (mM^−1^·cm^−1^) is the molar extinction coefficient of hydrazones and 0.65 is the absorbance of a standard protein, i.e., bovine serum albumin, 1 mg/mL in guanidine. All the reagents used were from Sigma-Aldrich. The percentage of reduction of carbonyl values in meat through the addition of collagen peptides solutions was determined with respect to the carbonyl value without peptides. 

### 4.6. Statistical Analysis and Experimental Data Modelling 

Analysis of variance (ANOVA) and multivariate analysis, namely principal components analysis (PCA) and hierarchical clustering analysis (HCA), were carried out with the software *STATISTICA v.13.3* (Tibco Software; Palo Alto, USA); confidence level was set at 95%. Modelling of antioxidant data (for FRAP and DPPH assays) was performed with the software *OriginLab v.9* (OriginLab Corporation, Northampton, USA); the goodness-of-fit was set at a 95% confidence level. 

## 5. Conclusions

Assessing the effect of biological parameters such as the age and the anatomy of collagen-rich tissues can promote a better application of collagen bioactive peptides. Furthermore, this can also help understanding the synergistic effects of collagen related and co-extracted compounds on the bioactivity. 

## Figures and Tables

**Table 1:** FRAP index (mean values) at 80 °C for each enzyme, expressed as mmol of electrons donated per 100 g of peptides solution. FO = old femur, 10 years; TO = old tibia, 10 years; FY = young femur, 4.5 years; TY = young tibia, 4.5 years.

	*Alcalase*	*Collagenase*	*Esperase*	*Neutrase*	*Papain*	*Savinase*
FO	0.0050 ^a,A^	0.0035 ^a,B^	0.0077 ^a,C^	0.0076 ^a,C^	0.0096 ^a,D^	0.0029 ^a,E^
TO	0.0051 ^b,A^	0.0073 ^b,B^	0.0084 ^b,C^	0.0075 ^b,D^	0.0140 ^b,E^	0.0083 ^b,F^
FY	0.0092 ^c,A^	0.0102 ^c,B^	0.0094 ^c,C^	0.0064 ^c,D^	0.0138 ^c,E^	0.0076 ^c,F^
TY	0.0136 ^d,A^	0.0130 ^d,B^	0.0090 ^d,C^	0.0110 ^d,D^	0.0178 ^d,E^	0.0104 ^d,F^

For each enzyme, significant differences among bones (*p* < 0.05) are indicated by different superscript lowercase letters; for each bone, differences among enzymes (*p* < 0.05) are indicated by different superscript capital letters.

**Table 2:** FRAP index at 80 °C for each enzyme, expressed as mmol of electrons donated per 100 g of peptides. FO = old femur, 10 years; TO = old tibia, 10 years; FY = young femur, 4.5 years; TY = young tibia, 4.5 years.

	*Alcalase*	*Collagenase*	*Esperase*	*Neutrase*	*Papain*	*Savinase*
FO	16.1 ± 0.8 ^a,A^	7.7 ± 0.5 ^a,B^	25.3 ± 0.9 ^a,C^	23.4 ± 2.1 ^a,D^	40.4 ± 0.9 ^a,E^	10.5 ± 1.3 ^a,F^
TO	11.2 ± 1.1 ^b,A^	15.0 ± 0.3 ^b,B^	29.3 ± 1.5 ^b,C^	17.9 ± 1.8 ^b,D^	25.9 ± 0.8 ^b,E^	18.3 ± 0.9 ^b,D^
FY	29.6 ± 1.3 ^c,A^	30.8 ± 1.2 ^c,A^	33.5 ± 1.7 ^c,B^	21.3 ± 1.6 ^c,C^	20.2 ± 1.4 ^c,D^	23.6 ± 1.2 ^c,E^
TY	40.2 ± 0.9 ^d,A^	37.0 ± 0.9 ^d,B^	32.9 ± 1.3 ^c,C^	30.1 ± 2.0 ^d,D^	25.2 ± 1.6 ^b,E^	23.0 ± 1.5 ^c,F^

For each enzyme, significant differences among bones (*p* < 0.05) are indicated by different superscript lowercase letters; for each bone, differences among enzymes (*p* < 0.05) are indicated by different superscript capital letters.

**Table 3:** DPPH• inhibition (%) per mg of peptides, at 80 °C. FO = old femur, 10 years; TO = old tibia, 10 years; FY = young femur, 4.5 years; TY = young tibia, 4.5 years.

	*Alcalase*	*Collagenase*	*Esperase*	*Neutrase*	*Papain*	*Savinase*
FO	69.0 ± 2.3 ^a,A^	35.8 ± 0.8 ^a,B^	57.3 ± 1.1 ^a,C^	72.8 ± 1.3 ^a,D^	80.2 ± 0.9 ^a,E^	60.6 ± 2.2 ^a,F^
TO	41.3 ± 1.5 ^b,A^	38.8 ± 1.6 ^b,B^	56.6 ± 0.6 ^a,C^	57.6 ± 0.8 ^b,C^	35.3 ± 2.3 ^b,D^	36.7 ± 1.4 ^b,E^
FY	48.0 ± 1.8 ^c,A^	57.3 ± 1.7 ^c,B^	66.7 ± 1.6 ^b,C^	78.5 ±1.6 ^c,D^	25.7 ± 1.4 ^c,D^	50.5 ± 1.3 ^c,E^
TY	47.3 ± 1.6 ^c,A^	52.1 ± 2.1 ^d,B^	59.5 ± 1.5 ^c,C^	66.1 ±1.4 ^d,D^	24.2 ± 1.8 ^d,E^	34.9 ± 1.8 ^d,F^

For each enzyme, significant differences among bones (*p* < 0.05) are indicated by different superscript lowercase letters; for each bone, differences among enzymes (*p* < 0.05) are indicated by different superscript capital letters.

**Table 4:** Reduction of carbonyls values (%) in 210 mg of meat protein (i.e., 1 g of raw meat), per mg of collagen peptides, at ambient temperature. FO = old femur, 10 years; TO = old tibia, 10 years; FY = young femur, 4.5 years; TY = young tibia, 4.5 years.

	*Alcalase*	*Collagenase*	*Esperase*	*Neutrase*	*Papain*	*Savinase*
FO	15.1 ± 2.1 ^a,A^	10.3 ± 0.7 ^a,B^	14.1 ± 1.3 ^a,C^	14.4 ± 0.6 ^a,C^	20.5 ± 1.1 ^a,E^	18.1 ± 0.7 ^a,F^
TO	9.6 ± 1.8 ^b,A^	9.4 ± 1.2 ^b,A^	15.6 ± 1.8 ^b,C^	9.5 ± 0.9 ^b,A^	7.7 ± 1.2 ^b,B^	9.9 ± 0.9 ^b,C^
FY	12.8 ± 1.6 ^c,A^	10.5 ± 2.0 ^a,B^	15.2 ± 0.9 ^b,C^	12.7 ±1.1 ^c,A^	6.0 ± 0.3 ^c,D^	11.2 ± 1.1 ^c,E^
TY	14.1 ± 1.3 ^d,A^	12.1 ± 0.9 ^c,B^	18.5 ± 1.3 ^c,C^	11.1 ±0.4 ^d,D^	7.1 ± 0.8 ^b,E^	8.6 ± 1.2 ^d,F^

For each enzyme, significant differences among bones (*p* < 0.05) are indicated by different superscript lowercase letters; for each bone, differences among enzymes (*p* < 0.05) are indicated by different superscript capital letters.

**Table 5:** Reduction of TBArs (%) in 22.5 mg of meat lipids (i.e., 1 g of raw meat), per mg of collagen peptides, at ambient temperature. FO = old femur, 10 years; TO = old tibia, 10 years; FY = young femur, 4.5 years; TY = young tibia, 4.5 years.

	*Alcalase*	*Collagenase*	*Esperase*	*Neutrase*	*Papain*	*Savinase*
FO	55.2 ± 1.8 ^a,A^	30.3 ± 0.9 ^a,B^	63.1 ± 2.2 ^a,C^	56.3 ± 2.1 ^a,A^	76.2 ± 1.2 ^a,D^	65.1 ± 1.4 ^a,E^
TO	33.4 ± 1.1 ^b,A^	22.5 ± 1.3 ^b,B^	66.2 ± 1.7 ^b,C^	39.4 ± 1.6 ^b,D^	34.1 ± 0.9 ^b,A^	38.2 ± 1.3 ^b,E^
FY	58.2 ± 1.6 ^c,A^	52.4 ± 1.6 ^a,B^	55.1 ± 1.4 ^b,C^	58.1 ± 1.4 ^c,A^	27.2 ± 0.8 ^c,D^	52.2 ± 1.2 ^c,B^
TY	47.1 ± 1.3 ^d,A^	43.3 ± 1.5 ^c,B^	53.2 ± 1.9 ^c,C^	50.2 ± 0.9 ^d,D^	25.3 ± 1.1 ^b,E^	44.1 ± 1.5 ^d,F^

For each enzyme, significant differences among bones (*p* < 0.05) are indicated by different superscript lowercase letters; for each bone, differences among enzymes (*p* < 0.05) are indicated by different superscript capital letters.

**Table 6:** Kinetics parameter for the iron reducing activity models (Equation (1a) and (1b)) and models adjustment (Adj. χ^2^) for papain and esperase peptides. FO = old femur, 10 years; TO = old tibia, 10 years; FY = young femur, 4.5 years; TY = young tibia, 4.5 years.

Peptides (FRAP)	*y*_0_ (µM Fe ^2+^)	*T*_0_ (°C)	*A* (µM Fe ^2+^)	*t* (°C ^−1^)	*Adj. χ^2^*
Papain FO *(ambient–70 °C)* (Equation (1a))	7.9	0	4.07	21.52	0.97
Papain TO (Equation (1a))	6.22	0	5.77	23.96	0.98
Papain FY (Equation (1a))	3.97	0	5.15	23.23	0.98
Papain TY (Equation (1b))	16.94	17.25	14.76	24.24	0.96
Esperase FO *(50–80 °C)* (Equation (1a))	50.37	0	4.07	17.58	0.95
Esperase TO (Equation (1a))	20.12	0	1.57	20.28	0.99
Esperase FY (Equation (1a))	20.28	0	1.96	20.71	0.96
Esperase TY (Equation (1a))	13.05	0	4.56	26.31	1
	*a* (µM Fe ^2+^)	*b* (µM Fe ^2+^/°C)			
Papain FO *(70–80 °C)* (Equation (2))	99	0.2			
Esperase FO *(ambient–50 °C)* (Equation (3))	−1.53	6.23			

**Table 7:** Kinetic parameter for the DPPH• quenching models and model adjustment (Adj. χ^2^) for neutrase and esperase. FO = old femur, 10 years; TO = old tibia, 10 years; FY = young femur, 4.5 years; TY = young tibia, 4.5 years.

Peptides (DPPH)	*y_0_* (µM Fe ^2+^)	*A* (µM Fe ^2+^)	*t* (°C ^−1^)	*Adj. χ2*
Esperase TY (Equation (1a))	10.42	0.57	19.02	0.98
Neutrase TO (Equation (1a))	1.99	8.14	36.97	0.99
Neutrase FY (Equation (1a))	9.86	3.42	27.70	0.96
Neutrase TY (Equation (1a))	10.10	5.38	32.81	0.99
Peptides (DPPH)	*a*	*b*	*c*	*Adj. χ2*
Esperase FO *(amb.–50 °C)* (Equation (4))	0.83	−4.88	−16.93	0.97
Esperase FO *(50–80 °C)* (Equation (5))	340	−0.27		0.99
Esperase TO *(amb.–50 °C)* (Equation (2))	0.80	0.47		1
Esperase TO *(50–80 °C)* (Equation (2))	−17.83	0.85		0.96
Esperase FY *(amb.–50 °C)* (Equation (2))	−1.58	0.48		0.98
Esperase FY *(50–80 °C)* (Equation (2))	−39.33	1.20		0.99
Neutrase FO *(amb.–50 °C)* (Equation (2))	5.54	0.47		0.98
Neutrase FO *(50–80 °C)* (Equation (5))	458.37	−0.27		0.99

**Table 8:** Composition of collagen powders. FO = old femur, 10 years; TO = old tibia, 10 years; FY = young femur, 4.5 years; TY = young tibia, 4.5 years.

Sample	Organic Matter (%)	Collagen (%)	Non-Collagen (%)	Minerals (%)	Calcium (%)
FO	35.3	34.5	0.83	64.7	20.5
TO	36	35.1	0.93	64	20
FY	39	38.4	0.64	61	19
TY	40	39.2	0.74	60	18

**Table 9:** Molecular weight, specificity, cutting sites, and optimal pH and temperature of hydrolysis for the 6 enzymes (endopeptidases) tested to hydrolyse bovine bone collagen (AA = amino acid).

Enzyme	MW (kDa)	Specificity/Cutting Sites	Temperature (°C)	pH
Alcalase	20–45	non-specific/larger and uncharged AA	60	7
Collagenase B	68–130	specific/Gly-AA	37	7
Esperase	20–30	non-specific/larger and uncharged AA	55	10
Neutrase	37	non-specific/larger and uncharged AA	55	6
Papain	23.4	specific/Ala, Val, Leu, Ile, Phe, Tyr	65	6.5
Savinase	20–30	non-specific/larger and uncharged AA	55	10

## Supplementary Materials

The following are available online: **Figure S1**. (A) Average width and length, and circularity of particles obtained by solubilising collagen powders in 0.1 mM acetic acid right before the addiction of the enzyme and at 3 mg/mL; (B) scanning electron microscopy (SEM) image of collagen powders; **Table S1.** Secondary structure of young and old bovine femur and tibia collagen solutions hydrolysed through the enzymes alcalase, collagenase, esperase, neutrase, papain, and savinase at the best Temperature and pH conditions for each enzyme; **Figure S2.** PCA loading plots to visualise correlations between amount of collagen peptides and collagen structure for alcalase (A) and collagenase (B).
