# Supplementary material for: Redox Potential and Antioxidant Capacity of Bovine Bone Collagen Peptides towards Stable Free Radicals, and Bovine Meat Lipids and Proteins. Effect of Animal Age, Bone Anatomy and Proteases—A Step Forward towards Collagen-Rich Tissue Valorisation
Source: Molecules. 2020 Nov 19;25(22):5422. doi: 10.3390/molecules25225422 (PMC7699565; doi:10.3390/molecules25225422)
Supplement: Supplementary file 1 [file molecules-25-05422-s001.pdf]

molecules

MDPI

Supplementary Materials

Redox Potential and Antioxidant Capacity of Bovine Bone Collagen Peptides Towards Stable Free Radicals, and Bovine Meat Lipids and Proteins. Effect of Animal Age, Bone Anatomy and Proteases – A Step Forward Towards Collagen Rich Tissues Valorization

Laurent Aubry, Claude De-Oliveira-Ferreira, Véronique Santé-Lhoutellier, and Vincenza Ferraro *

INRAE, QuaPA, 63122 Saint-Gènes-Champanelle, France; laurent.aubry@inrae.fr (L.A.) ; claude.ferreria@inrae.fr (C.D.-O.-F.) ; veronique.sante-lhoutellier@inrae.fr (V.S.-L.)

* Correspondence: vincenza.ferraro@inrae.fr

Abstract: Collagen antioxidant peptides are being widely studied. However, no research has paid attention to biological parameters such as the age and anatomy of collagen-rich tissues, which can determine a change in tissue structure and composition, and then in bioactivity. Moreover, only few research works have studied and assessed peptides antioxidant activity on the food matrix. This work aimed to investigate the effect of bovine's bone age and anatomy, and of six different enzymes, on the antioxidant activity of collagen peptides. Collagen was extracted from young and old bovine femur and tibia; six different enzymes were used for peptides' release. The redox potential, the quenching of stable free radicals, and the antioxidant capacity on bovine meat lipids and proteins was evaluated, under heating from ambient temperature to 80 °C. Age and anatomy showed a significant effect; the influence of anatomy becomes most important with age. Each enzyme's effectiveness toward age and anatomy was not the same. The greatest amount of peptides was released from young bones' collagen hydrolysed with papain. The antioxidant activity was higher at higher temperatures, except for meat proteins. Assessing the effect of age and anatomy of collagen-rich tissues can promote a better application of collagen bioactive peptides.

Keywords: collagen peptides; bovine bone; age; anatomy; antioxidant activity; by-products valorisation

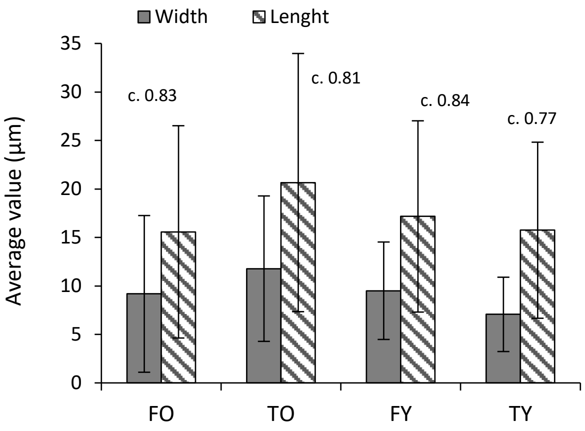

(A)

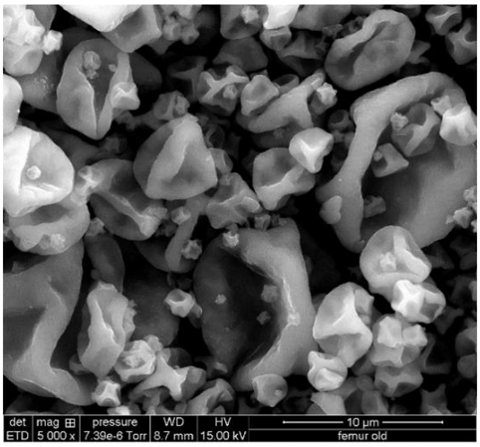

(B)

Figure S1. (**A**) Average width and length, and circularity (*c.*) of particles obtained by solubilising collagen powders in 0.1 mM acetic acid right before the addiction of the enzyme and at 3 mg/mL; (**B**) SEM image of collagen powders (example for FO). FO = old femur, 10 years; TO = old tibia, 10 years; FY = young femur, 4.5 years; TY = young tibia, 4.5 years.

Table S1. Secondary structure of young and old bovine femur and tibia collagen solutions hydrolysed through the enzymes alcalase, collagenase, esperase, neutrase, papain, and savinase at the best Temperature and pH conditions for each enzyme (mean values ± SD, *n* = 3). FO = old femur, 10 years; TO = old tibia, 10 years; FY = young femur, 4.5 years; TY = young tibia, 4.5 years.

	**37 °C (collagenase)**	**50 °C (neutrase)**	**55 °C (savinase/esperase)**	**60 °C alcalase**	**65 °C papain**
			Triple-helix (α-helix + β-turns + random) (%)		
FO	64 (43+13+0) ± 0.15	58 (31+4+23) ± 0.13	69 (49+20+0) ± 0.25	68 (47+21+0) ± 0.35	68 (49+19+0) ± 0.42
TO	63 (45+18+0) ± 0.21	66 (34+8+24) ± 0.15	38 (19+6+13) ± 0.36	66 (47+19+0) ± 0.11	67 (50+17+0) ± 0.17
FY	66 (46+20+0) ± 0.30	64 (36+19+9) ± 0.61	45 (21+10+14) ± 0.24	65 (45+15+0) ± 0.23	68 (49+19+0) ± 0.22
TY	72 (44+28+0) ± 0.24	66 (47+19+0) ± 0.68	64 (47+17+0) ± 0.12	68 (49+19+0) ± 0.28	71 (52+19+0) ± 0.23
			denatured 3-10 helix (%)		
FO	17 ± 0.23	18 ± 0.45	14 ± 0.36	11 ± 0.21	8 ± 0.71
TO	17 ± 0.41	18 ± 0.33	34 ± 0.41	12 ± 0.43	11 ± 0.11
FY	13 ± 0.44	14 ± 0.21	19 ± 0.31	14 ± 0.41	14 ± 0.53
TY	15 ± 0.35	15 ± 0.14	17 ± 0.32	14 ± 0.23	12 ± 0.21
			associated helices (%)		
FO	19 ± 0.46	23 ± 0.32	17 ± 0.4	21 ± 0.18	24 ± 0.18
TO	20 ± 0.21	16 ± 0.56	28 ± 0.21	22 ± 0.15	22 ± 0.17
FY	21 ± 0.53	22 ± 0.52	36 ± 0.23	21 ± 0.19	18 ± 0.42
TY	13 ± 0.23	19 ± 0.36	19 ± 0.38	18 ± 0.21	17 ± 0.15

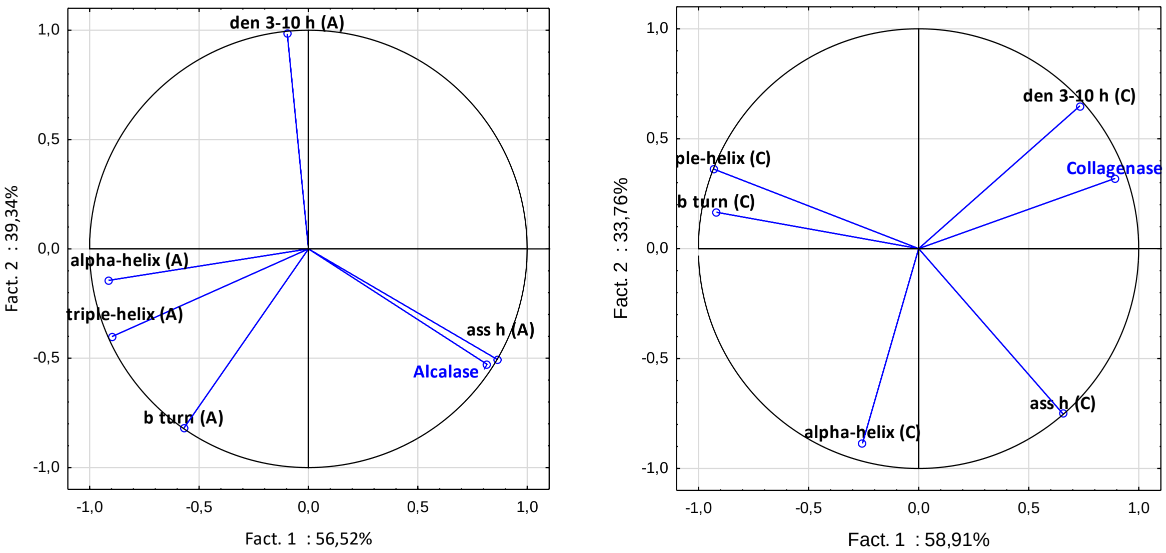

(A) (B)

Figure S2. PCA loading plots to visualise correlations between amount of collagen peptides and collagen structure for alcalase (**A**) and collagenase (**B**).

© 2020 by the authors. Submitted for possible open access publication under the terms and conditions of the Creative Commons Attribution (CC BY) license (http://creativecommons.org/licenses/by/4.0/).
